# Cytokines in gingival crevicular fluid in elderly rheumatoid arthritis patients in a population‐based cross‐sectional study: RANTES was associated with periodontitis

**DOI:** 10.1111/jre.12887

**Published:** 2021-05-05

**Authors:** Maria K. Söderlin, Gösta Rutger Persson, Stefan Renvert, Johan Sanmartin Berglund

**Affiliations:** ^1^ Department of Clinical Sciences Section of Rheumatology Lund University Lund Sweden; ^2^ Department of Periodontics, and the Department of Oral Medicine University of Washington Seattle WA USA; ^3^ Faculty of Health Sciences Kristianstad University Kristianstad Sweden; ^4^ Department of Health Blekinge Institute of Technology Karlskrona Sweden; ^5^ School of Dental Science Trinity College Dublin Ireland; ^6^ Faculty of Dentistry The University of Hong Kong Hong Kong SAR China

**Keywords:** cytokines, gingival crevicular fluid, periodontitis, rheumatoid arthritis

## Abstract

**Objective:**

We studied cytokines in gingival crevicular fluid (GCF) in a cross‐sectional population‐based cohort of rheumatoid arthritis (RA) patients ≥61 years of age with and without a diagnosis of periodontitis.

**Background data:**

Earlier studies on cytokines in GCF in RA patients have not given clear results.

**Methods:**

In a population‐based cross‐sectional study of patients ≥61 years of age, 233 RA patients were identified. 132 (57%) dentate RA patients participated. All participants received rheumatological and dental examinations, and had a panoramic radiograph taken. GCF was sampled on each patient. Interleukins 1‐β (IL‐1β), IL‐4, IL‐6, IL‐10, IL‐17A, tumor necrosis factor alpha (TNF‐α), interferon gamma (IFN‐γ), and chemokines RANTES/CCL5, eotaxin and monocyte chemoattractant protein (MCP‐1) were analyzed in GCF. These cytokines were stratified for periodontitis, age, gender, body mass index (BMI), smoking, and anti‐cyclic citrullinated protein (anti‐CCP) status. Binary logistic regression analyses with periodontitis as outcome were performed adjusting for the above mentioned confounding factors including anti‐rheumatic medication, disease duration and the cytokine in question.

**Results:**

Periodontitis was diagnosed in 80/132 (61%) of study participants. The 110 RA patients not participating were older, had a higher mean erythrocyte sedimentation rate (ESR), had a higher mean DAS28ESR (Disease Activity Score 28 using ESR) and were less often on biologic treatment. Only RANTES was associated with periodontitis (*p* = .049, OR 1.001, 95% CI 1.000–1.002) in the binary logistic regression analyses.

**Conclusion:**

In this population‐based elderly RA cohort, neither pro‐inflammatory nor anti‐inflammatory cytokines in GCF were clearly associated with a diagnosis of periodontitis.

## 1 INTRODUCTION

Rheumatoid arthritis (RA) and periodontitis are two chronic diseases that have been associated with elevated levels of circulating pro‐inflammatory cytokines and with clinical evidence of destruction of soft tissue and bone.^1^, ^2^ The inflammatory response in bacterially induced gingivitis and periodontitis results in gingival crevicular fluid (GCF) as an inflammatory exudate that can be collected at the gingival margin or within the gingival crevice or pocket. Since the 1960s, collection of GCF samples by a non‐invasive method has been used extensively to determine sites of active disease, assess disease progression and to monitor effect of periodontal therapy. Thus, a large number of cytokines, chemokines, bone remodeling enzymes, inflammatory mediators and tissue destruction enzymes in GCF can be used to assess periodontitis as a complement to the clinical evaluation.^3^, ^4^, ^5^ Several pro‐inflammatory and anti‐inflammatory cytokines interact and correlate with each other. The GCF cytokine levels can be influenced by the different GCF sampling techniques used (absorption, microcapillary, washing), analysing techniques and demographics, such as smoking, medications, and comorbidities.^6^, ^7^

Two recent literature reviews have summarized studies performed in GCF in RA patients on cytokines, chemokines, enzymes, and tissue breakdown products stratified for periodontitis.^8^, ^9^ There were inconclusive results as to levels of interleukin‐1β (IL‐1β) in periodontitis in RA as summarized from several cross‐sectional studies, and the same levels of interleukin‐4 (IL‐4) and interleukin‐10 (IL‐10) between RA patients, periodontitis, and periodontally healthy patients.^8^, ^9^ Matrix metalloproteinase‐8 (MMP‐8), MMP‐9, and tumor necrosis factor alpha (TNF‐α) levels were higher in periodontitis and in RA and MMP‐8 levels increased with the severity of periodontitis.^8^, ^9^ These reviews included very heterogeneous studies with mostly very small convenience samples of RA patients, where RA patients with systemic and comorbid conditions were excluded.

Several reviews and meta‐analyses of cytokines in GCF in otherwise healthy patients with periodontitis report that cytokines such as IL‐1α, IL‐1β, interleukin‐6 (IL‐6), GMCSF (granulocyte macrophage colony stimulating factor), interleukin‐12p40 (IL‐12p40), interleukin‐17 (IL‐17), TNF‐α, monocyte chemoattractant protein (MCP1/CCL2), matrix metalloproteases, bone remodeling products, prostaglandins, and macroglobulins are increased in periodontitis.^4^, ^5^, ^6^, ^10^, ^11^, ^12^, ^13^ Tomas et al showed that the best predictors for periodontitis included IL‐1α, IL‐1β, and IL‐17A in GCF.^11^

In a case‐control study, we have previously shown in this same RA patient cohort that RA was associated with a diagnosis of periodontitis with an odds ratio (OR) of 2.5 and that body mass index (BMI), periodontitis and female sex were associated with RA.^14^

We studied pro‐inflammatory and anti‐inflammatory cytokines and chemokines in GCF in RA patients who were 61 years of age or older in a systematically sampled cross‐sectional population‐based cohort, stratified for having or not having periodontitis as defined and described below. Our null hypothesis was that the expression of pro‐inflammatory cytokines in GCF from RA patients did not differ between those who had, or did not have, a clinical diagnosis of periodontitis. We were interested to study the cytokine and chemokine concentrations in GCF in a typical elderly RA patient population with long‐standing disease, comorbidities, and systemic conditions and prescribed anti‐rheumatic medication.

## 2 MATERIAL AND METHODS

**FIGURE 1:**
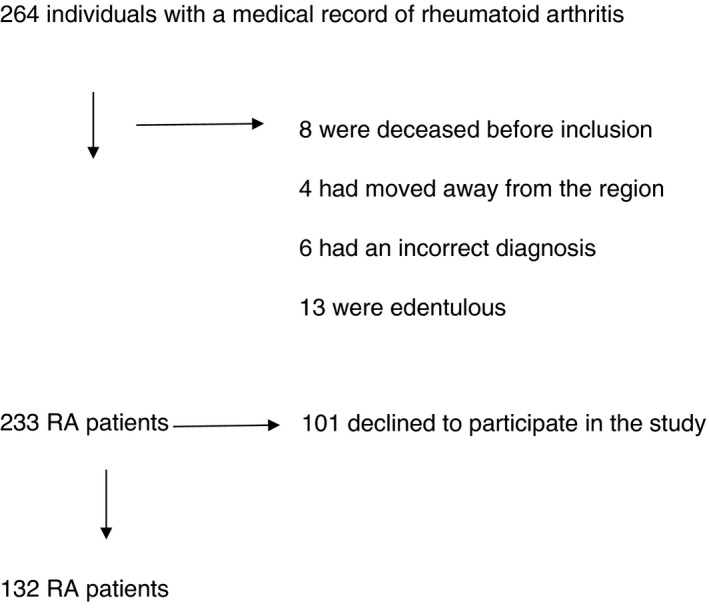
Study flowchart of identified and included participants

Between October 2013 and January 2015, all individuals with a diagnosis of rheumatoid arthritis (M05 and M06, International Classification of Diseases ICD‐10) found in their electronic medical records ≥61 years of age living in Karlskrona city in Sweden were identified. Data from all diagnosed patients with RA were available from the electronic regional database (Region Blekinge, Sweden). In the present study, participants were included if they consented to participate, and if they were diagnosed with RA, were ≥61 years of age per October 21, 2013, and living in Karlskrona city (population 64 000 in 2013). The RA patients were invited once to a rheumatological consultation at the outpatient clinic at the Rheumatology department per mail. The invitation included written information of the study.^14^ Figure 1 shows the study flowchart of identified and included participants.

All individuals with RA were examined at the outpatient rheumatology clinic by rheumatologists. The medical records were reviewed by a rheumatologist (author MKS). Data on RA disease activity and current anti‐rheumatic medications at inclusion were identified at the rheumatologists’ visit and the data were confirmed from the Swedish Rheumatology Quality Register online during the visit (www.srq.nu). Data on disease duration, previous anti‐rheumatic medications, comorbidities, osteoporosis, smoking habits (current, never, previous), occupation, BMI, the total number of drugs, and blood analysis including cholesterol levels, RF (rheumatoid factor) were recorded. We did not have data on previous antibiotic and/or previous periodontal treatment. Serum IgG antibodies to citrullinated peptides were analyzed by using a second generation cyclic citrullinated peptide (anti‐CCP) immunoassay (EuroDiagnostica) using the cut‐off limit (25 U/ml) recommended by the manufacturer, corresponding to the 99th percentile among healthy blood donors. Classification of the RA patients was performed according to the 1987 American College of Rheumatology (ACR) RA criteria^15^ and 2010 ACR/EULAR RA criteria.^16^ The RA diagnosis was made clinically. Disease activity was measured by the Disease Activity Score of 28 joints using ESR (DAS28ESR). DAS28ESR comprises of the number of swollen and tender joints of 28 joints (i.e., all joints in both arms, hands and knees), the patient's global assessment and ESR, and ranges from 0 to 10, 10 indicating maximum inflammation (www.das‐score.nl). We followed the STROBE guidelines for cohort studies.

### 2.1 Dental examination

A dental hygienist performed the clinical dental examinations. The examination also included a panoramic radiograph. Radiographs were assessed by a periodontist (author RGP) masked to clinical dental and medical data.

The following dental examination procedures were performed:

1. Measurements of probing pocket depths at four surfaces of teeth, and dental implants present.
2. The extent of bleeding on probing (BOP) was recorded within 30 s following probing of probing pocket depths (PPD) and assessed at the same sites as described above.
3. Dental plaque scores were recorded as described above.
4. Tooth decay was recorded as open decay.
5. The presence/absence of abnormal mucosal conditions was recorded.
6. The number of remaining teeth was registered and confirmed from the radiographs.
7. The extent of alveolar bone loss (mm distance between the cement‐enamel junction (CEJ) and bone level at interproximal sites) was assessed from panoramic radiographs. The proportions of sites with a depth ≥4 mm and ≥5 mm in relation to the number of assessed interproximal sites were calculated to arrive at a subject based number defining bone loss. The distances were assessed using digital images and the Osirix software version 9.0 (Pixmeo, SARL).

### 2.2 Definition of gingivitis and periodontitis

Periodontitis was defined as the clinical presence of bleeding on probing at >20% of recorded tooth surfaces, presence of >2 non‐adjacent sites with a PPD ≥5 mm, presence of bone loss at ≥2 sites with a distance between CEJ‐to bone level of ≥5 mm, or if evidence of a furcation invasion at molar teeth was found either clinically (grade II), or clearly visible on panoramic radiographs, and bone loss ≥5 mm at ≥30%. This definition of periodontitis was based on and adjusted to the current AAP/EFP classification of periodontitis ≥stage 2 to the extent possible due to differences in available data.

### 2.3 Sampling of gingival crevicular fluid

Samples of GCF were collected as follows. Contamination with saliva was prevented by the placement of a cotton roll in the mucosal fold at each test site and supra‐gingival plaque was removed with sterile cotton pellets. One sterile Perio Paper (Oraflow Inc) was placed into the mesial‐buccal pocket at each first molar tooth and was kept in situ for 30 s. The samples were then placed in labelled Eppendorf tubes (1.5 ml natural flat cap micro‐centrifuge tubes; Starlab). Within 30 min of sampling, vials were stored at −79°C until further analysis. Thus, four samples per study individual were placed in a vial. These four samples were pooled for the analyses. In the event a first molar in a quadrant was missing, the closest existing tooth was used for sampling.^17^ All sampling and clinical registrations were made by the dental examiner. All samples were examined at the University of Kristianstad bio‐medical laboratory. The mesio‐buccal aspect of first molars, or adjacent mesial tooth if a first molar was missing, was chosen as the specific test site for two reasons; (I) usually an easily assessable surface minimizing saliva contamination, (II) it was not possible to perform probing pocket depth measurements at a preceding visit. Collection of gingival fluid would not be valid if collected after probing. The volume of GCF was not assessed with a Periotron (Oraflow, Inc.) while uncontrolled loss of fluid/cytokine might occur. Instead, a 30 s standardized collection period was used to reflect the absorption of GCF volume.

### 2.4 Laboratory assays

The concentrations of IL‐1β, IL‐4, IL‐6, IL‐10, IL‐17A, TNF‐α, interferon gamma (IFN‐γ), and chemokines RANTES/CCL5, eotaxin and monocyte chemoattractant protein (MCP‐1) were determined in GCF samples using a commercial cytokine assay kit (according to the manufacturer's instructions for the xMAP technology using multiple beads and expressed as pg/ml (Bio‐Rad Laboratories). Plates were measured using the Bio‐Plex MagPix System and analyzed with the Bio‐Plex Manager (version 6.0; Luminex). In summary, the collected samples were thawed and suspended in 200 µl PBS containing 0.5% BSA. The vials were then centrifuged at 10 000 g for 10 min at 4°C. Antibody‐coupled magnetic beads were added to each 96‐well plate. The plates were washed with Bio‐Plex wash buffer (2100 µl). The peri‐implant GCF samples were then pipetted and distributed in duplicates. After 30 min of incubation, the samples were washed with buffer to remove unbound protein. A 25 µl aliquot of one concentration of Bio‐Plex biotinylated detection antibody specific for a different epitope of the cytokine was added to each well, incubated (30 min) and subsequently washed with Bio‐Plex wash buffer (3100 µl). The reaction mixture was detected by streptavidin‐PE (10 min), followed by a Bio‐Plex wash buffer (3100 µl). Beads were re‐suspended in each well with 125 µl of Bio‐Plex assay buffer, and shaken on a plate shaker (1100 rpm, 30 s). Cytokine concentrations in the samples were calculated by Bio‐Plex software using a standard curve derived from a recombinant cytokine standard, included in the 96‐well plate. Cytokine levels were assessed against reference curves for each cytokine according to guidelines (Bio‐Rad Laboratories).

### 2.5 Ethical approval

The Regional Ethical Review Board at Lund, Sweden, approved the study (LU 2013/323). All procedures performed in studies involving human participants were in accordance with the ethical standards of the institutional research committee of Lund University and with the 1964 Helsinki declaration and its later amendments or comparable ethical standards. Informed consent was obtained from all individual participants included in the study.

### 2.6 Statistics

Mann–Whitney test was used to study the association of cytokines and periodontitis, gender and anti‐CCP and the disease activity variables and demographics between periodontally healthy and periodontitis patients. Chi‐square was used for categorical variables. Spearman's correlation coefficient was used to study the correlation of cytokines, and to study the correlation of cytokines with age and BMI. Kruskal–Wallis test with Bonferroni correction was used to analyze the association of cytokines with smoking. We performed binary logistic regression analyses with the outcome of periodontitis using age, gender, smoking, disease duration, conventional synthetic DMARD treatment, biological treatment, glucocorticoid treatment, BMI, anti‐CCP, and the cytokine in question as predictive factors. Assessments of probing pocket depth values between two examiners performing clinical examinations using randomly selected cases from the study resulted in an intra‐class correlation of: 0.76 (95% confidence interval (CI) 0.67, 0.82; *p* < .001). The significance level was set at α < 0.05. Statistical analyses were performed using IBM SPSS Statistics version 25.

## 3 RESULTS

Data from 132 individuals with RA were analyzed, giving a catchment of 57%. The flowchart of identified and included participants is shown in Figure 1. The 110 RA patients who declined to participate in the study were older (74 vs. 70, *p* = .0001), had a higher mean erythrocyte sedimentation rate (ESR) (27 vs. 19, *p* = .0001), had a higher mean DAS28ESR (Disease Activity Score using ESR) (3.4 vs. 3.0, *p* = .005) and were less often on biologic treatment (7% vs. 22%, *p* = .003), but did not otherwise differ from the RA patients included in the study. A total of 83% of the RA patients fulfilled the 1987 ACR classification criteria and 72% the 2010 ACR/EULAR classification criteria for RA. The period prevalence of RA was 1.5%.

Table 1 shows the demographics, disease activity, anti‐rheumatic medication and comorbidities of the patients. Eighty out of the 132 (61%) study individuals were defined as having established/moderate/stage ≥2 periodontitis. There were no differences in the demographics, medications, and disease activity between patients having and not having periodontitis. Periodontitis patients had more often hypertension (70% periodontitis vs. 42% periodontally healthy, *p* = .002), but otherwise the comorbidities were evenly distributed between the two groups. A total of 89% of the patients had regular dental health care at least once a year or more often.

**TABLE 1:** Demographics, disease activity, medication, and comorbidities stratified for periodontitis. Mann–Whitney and chi‐square

Variable	*N*		No periodontitis	Periodontitis	*p* value
Age, years, mean (SD)	132	71 (6.6)	*N* = 52 69 (5.2)	*N* = 80 71 (7.2)	.111
Female %	132	71%	*N* = 52 69%	*N* = 80 71%	.804
BMI, mean (SD)	132	27 (5)	*N* = 52 27 (4.4)	*N* = 80 27 (5.4)	.418
Disease duration from symptom start, years, mean (SD)	127	14 (13)	*N* = 48 15 (15)	*N* = 79 14 (12)	.748
VAS pain, mm, mean (SD)	129	36 (28)	*N* = 51 38 (30)	*N* = 78 34 (27)	.505
ESR, mm, mean (SD)	129	19 (16)	*N* = 51 18 (16)	*N* = 78 20 (16)	.191
CRP, mg/ml, mean (SD)	132	9 (9)	*N* = 52 9 (9)	*N* = 80 9 (9)	.608
DAS28ESR classes, %	131		*N* = 51	*N* = 80	.052
DAS28 <2.6 remission		40.5%	49%	35%	
2.61–3.2 low disease activity		21.4%	9.8%	28.7%	
3.21–5.1 moderate disease activity		32.8%	33.3%	32.5%	
>5.1 high disease activity		5.3.%	7.8%	3.8%	
Anti‐CCP positive %	130	67%	*N* = 51 69%	*N* = 79 66%	.74
RF positive %	129	59%	*N* = 50 58%	*N* = 79 58%	.210
Number of current conventional synthetic DMARDs, mean (SD)	132	0.8 (0.6)	*N* = 52 0.8 (0.5)	*N* = 80 0.7 (0.6)	.633
Total number of all medications at inclusion, mean (SD)	132	10 (4.4)	*N* = 52 10 (4.5)	*N* = 80 10 (4.3)	.991
On conventional synthetic DMARDs	132	67%	*N* = 52 69%	*N* = 80 65%	.614
On methotrexate	132	57%	*N* = 52 50%	*N* = 80 61%	.202
On biologic treatment	132	22%	*N* = 52 23%	*N* = 80 21%	.804
On glucocorticoids	132	48%	*N* = 52 56%	*N* = 80 43%	.202
Smoking %	132		*N* = 52	*N* = 80	.239
Never smokers		37.9%	40.4%	36.3%	
Previous smokers		53%	55.8%	51.2%	
Current smokers		9.1%	3.8%	12.5%	
Socieconomic status, %	132		*N* = 52	*N* = 80	.314
Upper white collar		9.8%	15.4%	6.3%	
Lower white collar		35.6%	36.5%	35%	
Manual worker		50%	44.2%	53.8%	
Self‐employed		3.0%	3.8%	2.5%	
Housewife		1.5%	0	2.5%	
Diabetes type I and II, number (%)	132	18 (13.6%)	7 (13.5%)	11 (13.8%)	.962
Angina pectoris, number (%)	132	16 (12.1%)	5 (9.6%)	11 (13.8%)	.477
Acute myocardial infarction, number (%)	132	11 (8.3%)	5 (9.6%)	6 (7.5%)	.667
Stroke (embolus), number (%)	132	11 (8.3%)	4 (7.7%)	7 (8.8%)	.830
Hypertension, number (%)	132	78 (59.1%)	22 (42.3%)	56 (70%)	.002
Atrial fibrillation, number (%)	132	14 (10.6%)	8 (15.4%)	6 (7.5%)	.151
Transient ischaemic attack (TIA), number (%)	132	4 (3%)	2 (3.8%)	2 (2.5%)	.659
Coronary bypass operation, number (%)	132	4 (3%)	2 (3.8%)	2 (2.5%)	.659
Sjögren's syndrome, number (%)	132	3 (2.3%)	1 (1.9%)	2 (2.5%)	.686
Sicca, number (%)	132	5 (3.8%)	1 (1.9%)	4 (5.0%)	.366
Osteoporosis, number (%)	112	30 (22.7%)	*N* = 42 12 (23.1%)	*N* = 70 18 (22.5%)	.741
Epilepsy, number (%)	132	1 (0.8%)	0	1 (1.3%)	.418
Chronic obstructive pulmonary disease, number (%)	132	5 (3.8%)	2 (3.8%)	3 (3.8%)	.977
Interstitial lung disease, number (%)	132	6 (4.5%)	4 (7.7%)	2 (2.5%)	.162

Abbreviations: BMI, body mass index; VAS, visual analogue scale; ESR, Erythrocyte sedimentation rate; CRP, C‐ reactive protein; DAS28ESR, Disease Activity Score (28 joints) calculated with ESR; Anti‐CCP, Anti‐cyclic citrullinated protein; RF, Rheumatoid factor; DMARD, Disease‐modifying anti‐rheumatic drug.

### 3.1 Dental conditions

The RA patients had mean number of remaining teeth 22.0 (standard deviation (SD) 5.6). A total of 19.8% had <20 teeth remaining. Mean percent visible dental plaque scores in the RA patients was 31.3 (SD 24.7). If only individuals with ≥10 teeth remaining were considered the corresponding mean percent plaque scores was 31.5 (SD 23.9). The mean percent BOP was 21%.

### 3.2 Cytokine levels in GCF

Table 2 shows the levels (pg/ml) of the proinflammatory and anti‐inflammatory cytokines and chemokines in the 132 RA patients. Table 3 shows the levels of the cytokines (pg/ml) stratified for periodontitis. We could not see any differences between the cytokine levels stratified for periodontitis in any of the cytokines and chemokines studied (IL‐1β (*n* = 114, *p* = .234), IL‐4 (*n* = 104, *p* = .173), IL‐6 (*n* = 103, *p* = .223), IL‐10 (*n* = 123, *p* = .522), IL‐17A (*n* = 119, *p* = .731), INF‐γ (*n* = 124, *p* = .637), MCP‐1/CCL2 (*n* = 103, *p* = .247), eotaxin (*n* = 123, *p* = .613), TNF‐α (*n* = 103, *p* = .364) and RANTES/CCL5 (*n* = 103, *p* = .087)).

**TABLE 2:** Cytokine levels in GCF of the 132 RA patients. Pg/ml

Cytokine	Number	Mean (SD)	Median	Range	25th percentile	75th percentile
Interleukin‐1β	114	376.4 (328.6)	274.0	0–1861.4	145.2	532.8
Interleukin‐4	104	4.1 (1.7)	3.8	0.2–9.5	3.1	4.7
Interleukin‐6	103	6.8 (5.8)	4.8	0–33.1	3.6	9.0
Interleukin‐10	123	12.2 (16.6)	9.2	0–141.5	4.6	14.2
Interleukin‐17A	119	48.6 (37.2)	40.1	0–219.6	22.4	57.3
Interferon‐γ	124	20.9 (10.3)	19.0	3.65–73.63	15.8	21.5
MCP‐1	103	22.8 (13.9)	20.6	0–91.4	14.7	27.0
Eotaxin	123	6.6 (10.6)	5.0	0–116	3.0	6.8
TNF‐α	103	232.4 (147.4)	219.7	0–752.2	133.9	304.8
RANTES/CCL5	103	807.9 (1314.6)	398.4	0–8549.9	94.6	912.8

Abbreviations: MCP‐1, Monocyte chemoattractant protein; TNF, Tumor necrosis factor.

**TABLE 3:** Cytokine levels in GCF in the RA patients stratified for periodontitis. Pg/ml. Mann–Whitney

Cytokine	*N*	No periodontitis				*N*	Periodontitis				*p* value
		Mean (SD)	Median	25th percentile	75th percentile		Mean (SD)	Median	25th percentile	75th percentile	
Interleukin‐1β	48	304.7 (226.1)	274.0	128.0	382.0	66	428.5 (379.7)	278.1	146.2	646.4	.234
Interleukin‐4	44	3.9 (1.6)	3.8	2.7	4.5	60	4.3 (1.8)	4.0	3.2	5.0	.173
Interleukin‐6	44	6.3 (6.2)	4.3	3.3	8.1	59	7.2 (5.6)	5.9	3.9	10.3	.223
Interleukin‐10	50	9.8 (7.2)	8.0	4.6	13.7	73	13.9 (20.7)	9.8	4.6	14.4	.522
Interleukin‐17A	48	47.1 (34.8)	40.6	26.3	57.1	71	49.7 (39.0)	39.2	20.5	59.1	.731
Interferon‐γ	50	22.0 (12.9)	19.3	16.4	21.5	74	20.2 (8.1)	19.0	15.5	21.5	.637
MCP‐1	44	19.4 (7.8)	19.4	15.8	23.8	59	25.2 (16.7)	20.9	13.9	29.9	.247
Eotaxin	50	5.4 (3.6)	5.0	2.9	6.3	73	7.4 (13.4)	5.0	3.1	7.6	.613
TNF‐α	44	216.5 (140.8)	197.5	114.4	292.5	59	244.3 (152.2)	235.3	133.9	315.4	.364
RANTES/CCL5	44	464.2 (527.5)	327.3	83.7	592.5	59	1064.2 (1636.0)	409.1	144.0	1122.7	.087

Abbreviations: MCP‐1, Monocyte chemoattractant protein; TNF, Tumor necrosis factor.

All the cytokines correlated significantly with each other (*p* values between .000 and .012), with the exception of the levels of INF‐γ and IL‐1 β (*r*_s_ = 0.150, *p* = .112), INF‐γ and IL‐17A (*r*_s_ = 0.096, *p* = .297), and INF‐γ and RANTES (*r*_s_ = 0.076, *p* = .445).

### 3.3 Cytokines stratified for age, gender, smoking, anti‐CCP and BMI

Table 4 shows the cytokines stratified for age, gender, smoking, anti‐CCP status and BMI. There was an association between TNF‐α and age (*r*_s_ = 0.301, *p* = .002), MCP‐1 and age (*r*_s_ = 0.275, *p* = .005), IL‐10 and age (*r*_s_ = 0.222, *p* = .014) and RANTES and age (*r*_s_ = 0.344, *p* = .000), but not for the other cytokines and age. There was no association between gender, anti‐CCP antibodies and BMI and any of the cytokines. Interferon‐γ was associated with smoking (*p* = .006), where the association was between smoking and never smokers (*p* = .021) and previous smokers and never smokers (*p* = .035), where smokers had lowest levels of INF‐γ. There was an association of smoking with RANTES/CCL5 (*p* = .033), where the association was between smokers and previous smokers (*p* = .05), where smokers had lowest values. No association was found between smoking and the other cytokines.

**TABLE 4:** Cytokine levels stratified for age, gender, smoking, anti‐CCP status and BMI. Spearman's correlation coefficient, Kruskal‐Wallis and Mann‐Whitney

Cytokine	Number	Age	Gender	Smoking	Anti‐CCP antibodies	BMI
Interleukin1‐β	114	*p* = .071 *r*_s_ = 0.170	*p* = .214	*p* = .989	*p* = .139	*p* = .916 *r*_s_ = 0.010
Interleukin‐4	104	*p* = .084 *r*_s_ = 0.170	*p* = .256	*p* = .742	*p* = .848	*p* = .869 *r*_s_ = 0.016
Interleukin‐6	103	*p* = .283 *r*_s_ = 0.107	*p* = .523	*p* = .072	*p* = .190	*p* = .536 *r*_s_ = −0.062
Interleukin‐10	123	***p*** = **.014** ***r*_s_ = 0.222**	*p* = .115	*p* = .488	*p* = .454	*p* = .433 *r*_s_ = −0.071
Interleukin‐17A	119	***p*** = **.110** ***r*_s_ = 0.147**	*p* = .382	*p* = .364	*p* = .577	*p* = .709 *r*_s_ = 0.035
TNFα	103	***p*** = **.002** ***r*_s_ = 0.301**	*p* = .530	*p* = .430	*p* = .283	*p* = .781 *r*_s_ = 0.028
MCP‐1	103	***p*** = **.005** ***r*_s_ = 0.275**	*p* = .263	*p* = .094	*p* = .505	*p* = .774 *r*_s_ = −0.029
RANTES	103	***p*** = **.000** ***r*_s_ = 0.344**	*p* = .288	***p*** = **.033**	*p* = .302	*p* = .143 *r*_s_ = 0.145
Interferon‐γ	124	*p* = .077 *r*_s_ = 0.159	*p* = .831	***p*** = **.006**	*p* = .567	*p* = .071 *r*_s_ = −0.163
Eotaxin	123	*p* = .086 *r*_s_ = 0.155	*p* = .059	*p* = .281	*p* = .228	*p* = .799 *r*_s_ = −0.023

Note

The significant associations are highlighted

Abbreviations: MCP‐1, Monocyte chemoattractant protein; TNFα, Tumor necrosis factor alpha; anti‐ CCP, antibodies against cyclic citrullinated proteins; BMI, body mass index.

### 3.4 Binary logistic regression analyses

The binary logistic regression analyses showed that only RANTES was associated with periodontitis (RANTES *p* = .049, OR 1.001, 95% CI 1.000–1.002), but not the other cytokines analyzed (IL‐1β *p* = .164, IL‐4 *p* = .384, IL‐6 *p* = .273, IL‐10 *p* = .275, IL‐17A *p* = .588, MCP‐1 *p* = .066, TNFα *p* = .593, IFN‐γ *p* = .772 and eotaxin *p* = .529).

## 4 DISCUSSION

The data from the present cross‐sectional study of an elderly population‐based RA cohort failed to demonstrate any major differences in pro‐inflammatory and anti‐inflammatory cytokine levels in GCF stratified by having or not having periodontitis as defined in the present study. Only RANTES was weakly associated with periodontitis when adjusting for confounding factors. Thus, we retained our null hypothesis. Our study could not confirm earlier studies in healthy individuals and in RA patients that have shown a distinct pro‐inflammatory cytokine profile in patients with periodontitis.^4^, ^5^, ^6^, ^8^, ^9^

Studies on IL‐1β, a pro‐inflammatory cytokine that increases the production of chemokines and promotes the expression of metalloproteinases and prostaglandins, in GCF in RA patients have shown conflicting results.^18^, ^19^, ^20^, ^21^ Cetinkaya et al. showed that IL‐1β levels were comparable in RA patients and periodontitis patients, however, lower in periodontally healthy patients,^18^ and Biyikoglu et al showed that IL‐1β levels were comparable in periodontitis patient with and without RA.^20^ Miranda et al. showed that IL‐1β levels were higher in healthy patients,^19^ but Bender et al showed that IL‐1β levels were higher in RA patients.^21^ IL‐6, a pro‐inflammatory cytokine that increases the migration of inflammatory cells and promotes osteoclast activity, has been shown to have equal levels in GCF in RA patients with periodontitis and periodontitis patients without RA and periodontally healthy patients.^22^ TNF‐α, a pro‐inflammatory cytokine, has been shown to be the same in GCF in RA patients with periodontitis, periodontitis patients and healthy subjects,^23^ or increased in GCF and serum of RA patients and increased in periodontitis irrespective of RA status.^24^ Anti‐inflammatory cytokines IL‐4 and IL‐10 have been shown to be lower, or the same, in GCF in RA patients and periodontitis patients than in periodontally healthy controls.^18^, ^25^ Maldonado et al showed that MCP‐1 and IL‐17 were elevated and IL‐10 reduced in GCF in RA patients.^26^

These studies in GCF in RA patients are very heterogeneous and most have small sample size problems. Also, the studies have different study designs, recruitment processes, different definitions of periodontitis and different inclusion and exclusion criteria. The RA patients also have different anti‐rheumatic medications, if reported at all. The RA patient populations in these studies are not representative of typical RA patients due to multiple exclusion criteria, such as comorbid conditions, systemic diseases, previous antibiotic treatment and smoking. RA patients have both comorbid and systemic conditions and modern anti‐rheumatic treatment consists of multiple immunosuppression with the aim of remission. Most of these studies have also excluded patients receiving periodontal therapy. These differences in the studies make it very difficult to make direct comparisons and might explain the discrepancies in the results. It may also be that RA itself, the immunosuppressive medications used for RA, and chronic comorbidities and medications used to treat comorbid conditions may affect the production of cytokines in the gingival tissues in RA patients.

One of the strengths of our study was the systematically sampled population‐based cohort of elderly RA patients. Our RA patient cohort is representative of a general Swedish RA patient population ≥61 years of age. We included RA patients with comorbid and systemic conditions and smokers. Our RA patient population was well‐treated and stable with 62% of the patients being either in remission or having low disease activity. Because of the population‐based systematic sampling we could also make a RA prevalence calculation. We had data on patients declining participation in the study. The RA diagnosis was made clinically by rheumatologists. The demographics, medications, disease activity, and comorbidities were evenly distributed between patients having and not having periodontitis, except for more hypertension in the periodontitis group. It should be recognized that all study participants had access to government subsidized dental and medical care. A total of 89% of our RA patients had regular dental health care at least once a year or more often, and we did not exclude these patients. This may have influenced our results, as the GCF volumes and cytokine levels in well‐treated periodontitis may be low. The levels of dental plaque scores and BOP seemed to be similar to what has been reported earlier.^27^, ^28^, ^29^ The cross‐sectional nature of the present study did not allow assessments whether the study individuals had stable periodontal conditions or not.

Recently, the American Academy of Periodontology (AAP) and the European Federation of Periodontology (EFP) published new guidelines for the definition of periodontal conditions, and risk assessments.^30^ Over the last 20 years several attempts have been made to enhance the ability to diagnose and report on periodontal conditions.^30^, ^31^, ^32^, ^33^ The Swedish National Board of Health and Welfare still uses the criteria developed before 2018. The present study includes a study population from an ongoing national health survey that started in 2001 using criteria used at the time and that did not include assessments of clinical attachment levels or categories of risk. The ongoing NHANES (National Health and Nutritional Surveys) studies that have been in process from the 1960s also use older criteria for the definition of periodontal conditions. In the present study we have attempted to accommodate the most current perceptions of how to define periodontitis within the limits of data available and in compliance with the ethics approvals in effect. Obviously, this limits the extrapolation of data from our study with other studies using the most current but not yet fully evaluated 2018 diagnostic criteria. The other studies cited in the present report have used different models for the definition of periodontitis. This adds to the complexity in assessing the association and impact of periodontitis on RA conditions. In the present study, we considered the AAP/EFP current criteria for periodontitis. Based on our data set on the level of bleeding on probing, probing pocket depths, extent of radiographic evidence of bone loss, and tooth loss, both our definition and the criteria for the AAP/EFP definitions for stage II–IV periodontitis resulted in 80 cases.^30^ Thus, our composite index for a diagnosis of periodontitis is consistent with the current adapted classification system. Our study is a cross‐sectional study that does not allow identification of stable vs. progressive cases of periodontitis. It should be recognized that all study participants in the present study had access to all levels of dental care with insurance coverage and services at limited personal costs.

Due to the cross‐sectional study design, we cannot draw conclusions as to the effect of anti‐rheumatic medications on periodontitis, or the GCF cytokine levels. Studies on the effect of anti‐rheumatic medication on periodontitis would require a longitudinal follow‐up after treatment change. Longitudinal follow‐up studies of periodontitis and GCF would also mirror the chronic nature of both RA and periodontitis better. One weakness of the study is that we had a 43% drop‐out rate due to patients declining participation. The patients declining participation were older, had higher disease activity and were less often on biological treatment. A larger sample size would have given more power to the study. Also, we did not have a healthy control group. A large number of studies show that otherwise healthy periodontitis patients have a distinct pro‐inflammatory cytokine expression in GCF as compared to periodontally healthy persons.^4^, ^5^, ^6^, ^11^

In conclusion, we found no clear association of pro‐inflammatory or anti‐inflammatory cytokines in gingival crevicular fluid in elderly RA patients with periodontitis.

## CONFLICT OF INTEREST

The authors declare no conflict of interest.

## AUTHOR CONTRIBUTIONS

All authors contributed with substantial contributions to conception and design of, and acquisition of data and analysis and interpretation of data, drafted the article and revised it critically for important intellectual content and finally approved the version to be published.

## Data Availability

The data that support the findings of this study are available on request from the corresponding author. The data are not publicly available due to privacy or ethical restrictions.
